# Poxvirus Interactions with the Host Ubiquitin System

**DOI:** 10.3390/pathogens10081034

**Published:** 2021-08-16

**Authors:** Sian Lant, Carlos Maluquer de Motes

**Affiliations:** Department of Microbial Sciences, University of Surrey, Guildford GU2 7XH, UK; s.lant@surrey.ac.uk

**Keywords:** poxvirus, vaccinia virus, ectromelia virus, ubiquitin system, E3 ligases, Cullin-RING

## Abstract

The ubiquitin system has emerged as a master regulator of many, if not all, cellular functions. With its large repertoire of conjugating and ligating enzymes, the ubiquitin system holds a unique mechanism to provide selectivity and specificity in manipulating protein function. As intracellular parasites viruses have evolved to modulate the cellular environment to facilitate replication and subvert antiviral responses. Poxviruses are a large family of dsDNA viruses with large coding capacity that is used to synthetise proteins and enzymes needed for replication and morphogenesis as well as suppression of host responses. This review summarises our current knowledge on how poxvirus functions rely on the cellular ubiquitin system, and how poxviruses exploit this system to their own advantage, either facilitating uncoating and genome release and replication or rewiring ubiquitin ligases to downregulate critical antiviral factors. Whilst much remains to be known about the intricate interactions established between poxviruses and the host ubiquitin system, our knowledge has revealed crucial viral processes and important restriction factors that open novel avenues for antiviral treatment and provide fundamental insights on the biology of poxviruses and other virus families.

## 1. Introduction

Members of the *Poxviridae* family are large DNA viruses that infect a wide variety of species, from insects (classified in the *Entomopoxvirinae* subfamily) to vertebrates, including fish, reptiles, birds, and mammals (classified in the *Chordopoxvirinae* subfamily). The best-known poxviruses are variola virus (VARV), the smallpox agent, and vaccinia virus (VACV), its vaccine. In addition, several poxviruses are emerging zoonoses, like monkeypox virus (MPXV) and cowpox virus (CPXV), and others affect economically important animals such as orf virus and capripox viruses. Poxviruses follow a complex replication process in the cell cytosol, producing two types of virions generally known as extracellular virus (EV) and intracellular mature virus (MV) [1]. The poxvirus genome is a linear dsDNA of 150–300 kbp depending on the species. Whilst the replication of such a large DNA genome in the cell cytosol poses a unique challenge, it also offers an enormous coding capacity for a virus. Much of this coding capacity is devoted to manipulating the cellular environment and suppress the immune response elicited by viral infection and pathogen-associated molecular patterns (PAMPs) such as DNA and RNA [2,3]. A crucial cellular protein network that controls cellular responses both in homeostasis and under stress is the ubiquitin (Ub) system. The Ub system consists of an enzymatic cascade including E1 activating enzymes, E2 conjugating enzymes, and E3 ligases that, in combination, recognise cellular proteins and ubiquitylate them with single Ub moieties or poly-Ub chains [4,5]. Ubiquitylation is a covalent modification that alters the fate of the target protein, either its localisation, signalling properties or half-life. With >600 E3 Ub ligases, the Ub system provides a highly selective and powerful mechanism for protein targeting that many viruses including poxviruses have evolved to hijack. On the other hand, the Ub system constitutes a host defence mechanism to eliminate viral proteins and signal antiviral responses. This manuscript reviews recent advances in the multiple ways poxviruses interact with the Ub system, including the role of the proteasome in virus uncoating, the many interactions with the Cullin (Cul) family of E3 Ub ligases, the presence of Ub ligases and Ub-related genes in poxvirus genomes, and the ubiquitylation of viral proteins. Ub belongs to a larger family of structurally similar proteins that are also covalently attached, such as small Ub-like modifiers (SUMO), interferon stimulated gene 15 (ISG15), or neural-precursor-cell-expressed developmentally regulated 8 (NEDD8). Poxvirus interactions with these Ub-like families are not described in this manuscript. For earlier reviews on the topic, please see [6,7,8].

## 2. The Ubiquitin System

Ubiquitylation consists in the attachment of a Ub moiety onto a target protein via an isopeptide bond. In the first step, the activating E1 enzyme forms a thioester bond between the C-terminal glycine residue of Ub and a conserved cysteine residue of the E1, a process that requires ATP. The activated Ub is then transferred to the active site of the E2 conjugating enzyme, which acts as a carrier protein. Finally, Ub is linked to the substrate protein via its C-terminal glycine residue, a process facilitated by an E3 ligase [4,5]. Ubiquitylation generally occurs on the ε-amino group of a substrate lysine residue, but non-canonical ubiquitylation on alternative residues has also been reported [9]. Deubiquitinases (DUBs) can hydrolyse the isopeptide bonds established between Ub and a substrate, meaning that ubiquitylation of proteins is reversible. Furthermore, the process of ubiquitylation can create poly-Ub chains in the same manner between Ub molecules, as Ub itself contains seven lysine residues besides its starting methionine. This results into a remarkably complex range of poly-Ub chains, from short chains of just two molecules to long and branched chains formed of over ten moieties. In addition, both mono-Ub and poly-Ub can occur independently in different acceptor sites in the same protein, adding further complexity to the system. There are two main families of E3 enzymes: the ‘really interesting new gene’ (RING) E3 ligases, which mediate ubiquitylation by bringing the Ub-loaded E2 in close proximity to the substrate, and the ‘homologous to the E6AP carboxyl terminus’ (HECT) E3 ligases, which accept the Ub moiety from the E2 first before transferring it to the substrate.

Ubiquitylation can result in eight different types of linkage chains using either the starting methionine (M1), or any of the seven lysine residues in the Ub molecule (i.e., K6, K11, K27, K29, K33, K48, and K63) [4]. The first described and most extensively studied role for ubiquitylation is to target proteins for degradation by the proteasome, a process that most often involves K48-linked Ub chains. The 26S proteasome is a large protein complex found in eukaryotic cells that recognizes ubiquitylated proteins using a number of intrinsic receptors containing Ub-binding domains [10,11]. The Ub-tagged polypeptides are deubiquitylated and unfolded before being degraded. Thus, together with autophagic routes, the Ub-proteasome system (UPS) represents the major cellular mechanism for intracellular protein and organelle quality control [10,11]. Our knowledge on the roles of the other Ub chains has expanded extensively over the last years, and it is now generally recognized, for instance, that K63 and M1 linkages regulate intracellular signalling, including inflammation, DNA repair, or membrane protein trafficking, or that K11 chains impact on protein degradation and regulate mitotic exit (recently reviewed in [12,13]).

## 3. The Role of the UPS in Poxvirus Uncoating and Replication

The UPS is exploited by many viruses to manipulate host responses, promote viral replication, and progress in their life cycle [14,15,16]. Poxvirus infection initiates after membrane fusion and liberation of the nucleoprotein core into the cell cytosol. This is followed by genome release (generally known as uncoating) and replication. Whilst intermediate and late genes are post-replicative, early gene expression can occur within intact cores and is required for effective uncoating [17,18,19]. Early work demonstrated that the UPS is essential for orthopoxvirus (OPXV) infection since proteasome inhibitors showed complete suppression of viral DNA replication and late gene expression [20,21]. Proteasome inhibition did not affect virus entry nor viral replication when added post-infection, indicating that proteasomes are needed at an early stage of viral infection [20,21]. Subsequently, a genome-wide RNAi screen demonstrated the need for proteasome activity to break viral cores [22]. These incoming cores were found to be ubiquitylated with K48 chains, so proteome activity on the cores was independent of de novo ubiquitylation [22]. The presence of Ub in the viral cores is consistent with earlier works reporting the protein composition of poxvirus particles [23,24,25]. In addition to this, the UPS was also required to license the viral genome for replication. This second event required de novo ubiquitylation and participation of a cellular Cul-3 based Ub ligase [22], although the components that are ubiquitylated remain unknown.

The process of uncoating also requires viral proteins. A similar RNAi screen against 80 VACV proteins identified protein D5 as the main viral uncoating factor [26]. D5 is a highly conserved essential protein with ATPase and primase activities [27,28]. D5 is an early gene, and after its translation in the cytosol, it associates back with incoming cores in the absence of UPS activity [26], indicating that it has a direct role in core breakdown and genome release. There are no structural domains in D5 that suggest an association with Ub and the UPS, so how D5 operates remains to be elucidated. More recently, the viral proteins C5, M2, and 68k-Ank (equivalent to B18 in the VACV Copenhagen nomenclature) were also found to be involved in uncoating and DNA replication [29]. Engineered modified vaccinia virus Ankara (MVA), lacking 68k-Ank, was deficient for uncoating as well as, independently, DNA replication [29,30]. These phenotypes were reversed by expression of either C5 or M2, which revealed functional redundancy in the viral control of viral uncoating and DNA synthesis [29]. Interestingly, 68k-Ank contains an F-box domain that allows interaction with host Cul-1 complexes [31], whereas C5 contains a BTB domain that mediates interaction with host Cul-3 [7]. The presence of these domains suggests direct associations with the Cul family and the UPS, although a 68k-ANK lacking the F-box domain retained post-replicative gene expression [30]. Taken together, these studies have revealed the increasing complexity of the intertwined processes of uncoating and DNA synthesis. Identification of the core components that are ubiquitylated upon core release as well as during virion assembly and the cellular machinery responsible for these activities will aid in the discovery of novel viral targets and the development of antiviral drugs.

## 4. Viral Interaction with the Cul-RING E3 Ub Ligases

### 4.1. Functional Architecture of Cul-RING Ub Ligases

**Figure 1:**
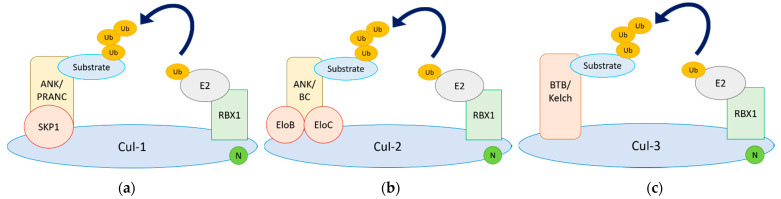
Architecture of Cullin-RING E3 Ub ligases (CRLs) and its manipulation by poxvirus adaptors. Representative complexes for Cul-1-, Cul-2-, and Cul-3-based complexes are shown. In each case, a Cullin (Cul) molecule acts as a scaffold bridging the E2 conjugating machinery with a substrate recruited by a substrate receptor/adaptor. Poxviruses hijack these complexes by encoding their own substrate receptor. Poxviruses encode (**a**) Ankyrin proteins containing PRANC domains (pox protein repeat of ANK C-terminus) to hijack CRL1; (**b**) Ankyrin proteins containing BC boxes to hijack CRL2; and (**c**) BTB/Kelch proteins to hijack CRL3.

Cul-RING Ub ligases (CRLs) constitute the largest family of Ub ligases and account for at least one-fifth of proteasome-dependent degradation within cells [32]. Each CRL utilises a unique Cul protein that acts as a scaffold for the formation of a multi-protein complex able to transfer Ub from an E2 conjugating enzyme onto a substrate protein [33]. The Cul family includes 7 Cul proteins (Cul-1, -2, -3, -4A, -4B, -5, and -7), plus the non-canonical anaphase-promoting complex/cyclosome (APC/C) and p53 cytoplasmic anchor protein (PARC) that contain Cul homology domains [33]. The C terminus of Cul associates with a RING protein, whereas the N terminus recruits the substrate protein via a subset of substrate receptors and adaptors. Multiple substrate receptors associate with each CRL core, thereby increasing the repertoire of substrates that can be ubiquitylated. In addition, many substrates are post-translationally modified prior to ubiquitylation, ensuring that only the modified pool of the total substrate population is targeted [34]. The overall architecture of the complex is further regulated by neddylation (which activates Ub ligase activity) and deneddylation of Cul [35,36]. The specificity and selectivity of CRL activity is therefore tightly regulated in agreement with the important functions these E3 Ub ligases have in development, cell cycle, signal transduction, transcription, and DNA repair [33]. These properties also make CRLs ideal targets for viruses, either to block ubiquitylation activity or to redirect it against antiviral molecules. We describe here the known interactions between poxvirus proteins and the Cul family and, when known, the corresponding implications (Figure 1).

### 4.2. Viral Cul-1 Adaptors

Cul-1-based CRLs (CRL1) utilise the adaptor Skp1 to recruit a variety of substrate receptors containing an F-box motif. CRL1s are thus commonly known as SCF ligases (for Skp1-Cul1-F-box) [37]. The F-box motif was originally described in cyclin F, but it is now recognised in >50 proteins found in a wide variety of eukaryotes [38]. Cul-1 can therefore associate with multiple different F-box proteins, and this process is known to be controlled by the Cul-binding protein CAND1 [39]. One of the largest families of proteins encoded by poxviruses encompasses Ankyrin-repeat proteins (ANK) [40]. ANK are common in eukaryotes, but not viruses. In addition, a significant proportion of poxvirus ANK are fused to a C-terminal sequence that resembles the cellular F-box domain [40,41,42]. Poxvirus ANK contain between 4 to 10 Ankyrin repeats in the N terminus and are conserved in most poxvirus genera, including Avipoxvirus, Parapoxvirus, Leporipoxvirus, Capripoxvirus, and OPXV [42]. The poxviral F-box domain (also known as pox protein repeat of ANK C-terminus or PRANC) is shorter than its cellular counterpart, and in most cases, it has two α helices as opposed to three in the mammalian F-box [43]. Despite this, it fulfils the same function in that it mediates interaction with CRL1 machinery replacing the cellular F-box adaptor (Figure 1a), and it can conjugate Ub chains [44,45,46]. Interestingly, the poxvirus F-box domain is only found in association with ANK. This combination does not actually exist in mammalian genomes, so it seems to have originated in an ancestral poxvirus and been subsequently retained and duplicated [40,42,47].

The number of ANK/F-box in poxviruses seems to vary largely. For instance, whilst molluscum contagiosum virus (MCV) encodes none, avipoxviruses such as fowlpox virus (FPXV) or canarypox virus (CNPV) encode at least 20. The function of most of these avipoxvirus ANK/F-box remains unknown, but some have been shown to block the induction of type I interferon (IFN-I) or to suppress the antiviral effects of IFN-I [48,49]. Within the parapoxvirus, orf virus encodes 5 ANK/F-box, all of which interact with CRL1 [44]. In addition, they all interact with FIH, the factor that hydroxylates the hypoxia-inducible factor (HIF) and reduces its transcriptional activity [50]. Despite the presence of a functional F-box domain, viral targeting of FIH did not trigger its degradation, but resulted in derepression of HIF responses, thereby acting as competitive inhibitors [50]. Interestingly, VACV induces a similar hypoxic-like state by an alternative mechanism that involves targeting HIF prolyl hydroxylases [51], revealing convergent viral evolution to achieve HIF activation.

The leporipoxvirus myxoma virus (MYXV) encodes four ANK/F-box proteins: M-T5, M148, M149, and M150. Deletion of all four proteins resulted in a robust activation of innate immune pathways, particularly the nuclear factor κ-light-chain-enhancer of activated B cells (NF-κB), and severe attenuation in rabbits [52]. M-T5 was the first ANK/F-box protein to be shown to interact with CRL1 complexes [46]. In addition, M-T5 interacts and activates the cellular kinase Akt [53,54]. Functionally, these interactions allow M-T5 to protect MYXV-infected cells from virus-induced cell cycle arrest and mediate MYXV tropism to cancer cells [46,53]. In contrast to M-T5, much less is known about the interaction partners and functions of M148, M149, and M150. The latter has been shown to associate with CRL1 and localise in the nucleus with NF-κB [55,56], suggesting that this family of proteins operates to suppress NF-κB function.

Within OPXV, CPXV contains the largest suite of ANK with 15 orthologue groups, in line with its larger genome and coding capacity [40,47,57]. Within these, 11 orthologue groups are ANK/F-box, 2 contain a BC-box domain instead of an F-box and associate with Cul-2 (ANK/BC), and 2 contain only Ankyrin repeats (ANK-only) [57]. In many cases, these orthologues are deleted or fragmented in other OPXV species. The conservation of these ANK orthologue groups within the OPXV is shown in Table 1. The molecular partners and functions of some of these ANK/F-box proteins have been elucidated in recent times. Cowpox virus CP77 is a host-range factor that, together with the ANK proteins K1 and C7, determines infectivity in different cell lines. For instance, VACV lacking both K1 and C7 fails to replicate in many mammalian cell lines, but this can be rescued by expression of either K1, C7, or CP77 [58,59]. Recent work has demonstrated that these three proteins target the sterile alpha motif domain-containing 9 (SAMD9) and its close paralogue SAMD9L, two IFN-stimulated genes (ISG) capable of restricting poxvirus replication [60,61,62,63]. In addition, CP77 has also been shown to suppress the activation of NF-κB by targeting the NF-κB subunit p65 through its first six ANK repeats [64].

The role of poxviral F-box proteins as immune modulators have also been documented in other OPXV. For instance, ectromelia virus (ECTV) encodes four ANK/F-box proteins able to suppress NF-κB activation [45,65]. One of these, ECTV 002, is an orthologue of CPXV 006 and is conserved in the pathogenic variola virus (VARV) and monkeypox virus (MPXV), but not in VACV [66]. CPXV 006 interacts with CRL1 and NF-κB1 and prevents NF-κB activation, suppressing host inflammatory responses and contributing to virulence [66,67]. More recently, CPXV 006 has also been shown to suppress virus-induced inflammation by targeting the necroptosis adaptor receptor-interacting protein kinase 3 (RIPK3) and thereby termed viral inducer of RIPK3 degradation or vIRD [68]. Necroptosis is an inflammatory cell death pathway that is activated when caspase activity and the apoptotic pathways are compromised. Poxviruses suppress apoptotic cell death very efficiently, employing a variety of different strategies (reviewed recently in [69,70]) and thereby sensitising the cell for necroptosis. vIRD uses its ANK repeats to target the RHIM domains of RIPK3 and trigger its CRL1-mediated degradation [68]. Thus, vIRD allows some OPXV to counteract the cellular necroptotic response and enhance viral replication whilst limiting antiviral inflammation. Finally, research with VACV has revealed that the ANK/F-box protein C9 antagonises the host IFN response by inducing the proteasomal degradation of IFN-induced proteins with tetratricopeptide repeats (IFITs) [71,72]. IFIT proteins recognise non-self mRNA, either uncapped or partially methylated capped, blocking its translation [73]. Given that VACV mRNA are fully capped, viral targeting of IFITs may reveal other antiviral functions by this family of cellular proteins.

### 4.3. Viral Inhibitors of CRL1 Function

**Figure 2:**
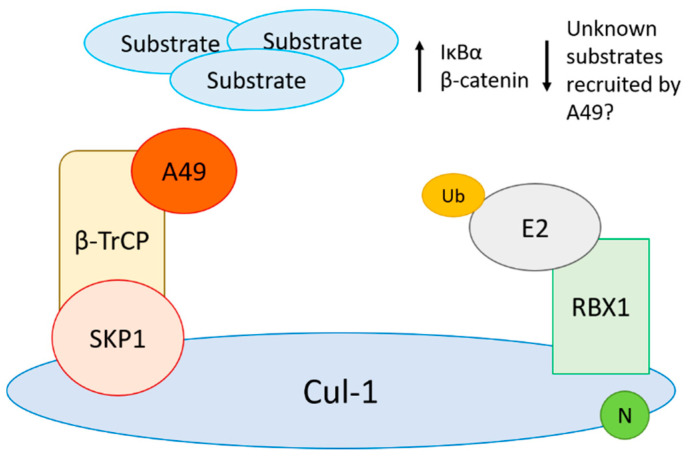
VACV protein A49 binds to and blocks β-TrCP function resulting in the absence of substrate recognition and ubiquitylation. This causes the accumulation of substrates such as IκBα and β-catenin, even in their phosphorylated forms. Poxviruses may also utilise A49 to redirect ubiquitylation towards unknown substrates, as seen with other viruses such as HIV.

Poxvirus interplay with the NF-κB signalling pathway is remarkable and involves multiple strategies (reviewed in [2,74]). In the NF-κB signalling cascade, the IκBα kinase (IKK) complex phosphorylates IκBα at Ser 32 and 36, allowing recognition by a CRL1 complex containing the F-box β-transducin repeat containing protein (β-TrCP), which catalyses ubiquitylation of the upstream K21/22 and triggers IκBα degradation [75]. As a consequence of this process, the NF-κB heterodimer is released and translocates into the nucleus. Despite the identification of many viral products affecting NF-κB activation, evidence existed that several poxviruses are able to suppress the degradation of the inhibitor of κB (IκB)α even in its phosphorylated form [65,76,77]. Research with VACV demonstrated that this phenomenon is mediated by protein A49, a protein conserved in several OPXV including VARV that blocks CRL1^β-TrCP^ function (Figure 2) [78,79]. A49 mimics the IκBα degron sequence in its N terminus, which protrudes out of the B-cell lymphoma (Bcl)-2-like core of the protein [78,80]. Upon signal transduction, IKKβ phosphorylates A49 which, in turn, binds β-TrCP, blocking its ability to recognise substrates such as IκBα and β-catenin [78,81,82]. A49 escapes CRL1^β-TrCP^-mediated degradation because it lacks the lysine acceptor sites typically present in β-TrCP substrates [78,80]. Whether A49 redirects ubiquitylation towards other substrates as observed, for instance, for the human immunodeficiency virus (HIV) protein Vpu, remains to be determined. Given the importance of certain CRLs, other viral proteins acting as direct inhibitors of CRL complexes, such as in the case of A49, may exist.

### 4.4. Viral Cul-2 Adaptors

Recent work has shown that some OPXV ANK proteins associate with cellular Cul-2-based CRL (CRL2) complexes [57]. CRL2 utilise ElonginB/C adaptor complex, which bind to the so-called BC-box or VHL-box (from the prototypic Cul-2 interactor von Hippel–Lindau or VHL protein) [83,84]. Viral CRL2-interacting proteins contain a BC-box domain (instead of an F-box) that allows association with Cul-2 and are termed ANK/BC (Figure 1b). The BC-box locates in the C terminus and similar to the viral F-box [43], it is shorter and retains only the most critical residues to mediate binding [57]. The reduced sequence also explains the absence of a Cul-5 box, which is sometimes identified in cellular adaptors [83,85]. The architecture of the complex is thus very similar to cellular CRL2 and suggests that the ANK domains in the ANK/BC proteins mediate protein–protein interactions aimed at triggering protein ubiquitylation. Although the identity of these ubiquitylated substrates remains undetermined, it is likely that they are involved in optimal innate immune activation as viral ANK/BC proteins were found to potently suppress NF-κB and IFN-I responses [57]. Interestingly, although the combination of ANK repeats and F-box domains is unique among viruses to poxviruses and has not been observed in mammalian genomes, cellular CRL2 complexes can associate with cellular ANK-containing adaptors [85]. This indicates that horizontal gene transfer from the host may be the origin of the viral ANK/BC, independently of the ANK/F-box, with ANK/BC being less widely distributed across poxvirus genera.

### 4.5. Viral Cul-3 Adaptors

Whilst CRL1 and CRL2 utilise adaptors and substrate receptors, Cul-3-based complexes (CRL3) differ and employ a single protein combining both functions. This type of adaptor therefore contains a domain that mediates interaction with Cul-3 known as bric-a-brac, tramtrack, and broad complex transcription (BTB) [86] or poxvirus and zinc finger (POZ) domain [87], and a domain of a different nature where the substrate binding function resides [88], overall yielding a complex structurally analogous to CRL1 and CRL2 (Figure 1c). Several poxvirus genera, including OPXV, leporipoxvirus, or capripoxvirus, encode BTB-Kelch proteins, which contain a variable number of Kelch repeats arranged into a single β-propeller. For instance, ECTV encodes 4 BTB-Kelch proteins, two of which (ECTV 150 and 167) have been shown to associate with Cul-3 and the ubiquitylating machinery via their BTB domain [89]. The orthologues of BTB/Kelch proteins within OPXV can be viewed in Table 2. VACV is predicted to encode 3 BTB-Kelch, namely C2, F3, and A55, although a fourth gene C5 contains a BTB domain. The VACV gene A55R is the orthologue of ECTV 150 and has recently been crystallised in complex with the N terminus domain of Cul-3 [90]. This structural complex has confirmed that the overall interaction and binding interface between viral and cellular BTB and Cul-3 is similar. Surprisingly, the affinity of the viral complex was stronger, providing a mechanism for how the virus hijacks CRL3 complexes during infection. Although the specific functions of most poxviral BTB-Kelch proteins remain to be elucidated, it has been shown that these proteins contribute to virulence and drive pathogenesis. Recombinant VACV engineered to lack C2, F3, or A55 showed alterations in the outcome of infection [91,92,93]. Similarly, deletion of the sheeppox virus BTB-Kelch protein 019 [94] or deletion of 4 BTB-Kelch proteins in CPXV [95] resulted in marked attenuation. At the cellular level, absence of C2, F3, or A55 during VACV infection or 019 during SPPV infection resulted in loss of Ca2+-independent cell adhesions [91,92,93,94]. This suggests that these proteins may modulate cellular adhesion. In the case of A55, it has recently been shown that this protein targets host importin-α, preventing binding and nuclear translocation of p65, thereby inhibiting NF-κB activation [96]. This is in agreement with a previous report revealing that ECTV 150 reduced NF-κB activation downstream of IκBα degradation [97]. Similar to other VACV NF-κB inhibitors [98,99,100,101,102], infection of mice with VACV lacking A55 resulted in elevated CD8+ T-cell memory and increased protection from challenge [96].

### 4.6. Targeting of Other CRLs

The molecular and functional architecture of CRL1, 2, and 3 is generally shared with Cul-4-based CRL (CRL4) and Cul-5-based CRL (CRL5). CRL4 can be formed by Cul-4A or Cul-4B, and in both cases employ the DDB1 adaptor and a large repertoire of substrate receptors known as DCAF (DDB1-Cul-4A-associated factors) [103,104]. It is predicted that at least 90 different DCAF can associate with CRL4, thereby providing high versatility to this E3 Ub ligase family [105]. Although several viral factors, including HIV protein Vpr, have been shown to manipulate CRL4 complexes [14,15,16], there are at present no reported poxvirus proteins targeting CRL4. On the other hand, CRL5 complexes are closely related to CRL2. They both utilise the ElonginB/C adaptors and substrate receptors containing a BC-box although, in this case, they also contain a SOCS-box that allows interaction with Cul-5 [83]. Molluscum contagiosum virus (MCV), the only extant human-specific poxvirus, targets CRL5 via protein MC132 [106]. MC132 binds CRL5 and p65 and induces the proteasomal degradation of the latter, thereby suppressing NF-κB activation [106]. MC132 is unique to MCV and has no recognised orthologs in other poxvirus genomes, and as such, it remains the only poxviral protein known to hijack CRL5 machinery.

In addition to CRL1-5, non-canonical Cul such as Cul-7, APC/C, and PARC exist and have been shown to have E3 Ub ligase activity [107,108,109,110]. The APC/C is a large multisubunit complex that is critical in regulating cell cycle progression and mediating ubiquitylation of mitotic cyclins. Amongst the many subunits, APC2 contains a distant Cul domain and acts as a scaffold, whilst APC11 contains a RING-like domain and associates with the E2 Ub-conjugating machinery to allow ubiquitylation of substrates recruited by a variety of substrate receptors [111]. Several poxvirus genera including parapoxviruses, molluscipoxviruses, crocodilepoxviruses, and the unclassified squirrelpox virus encode a homolog of APC11 [112]. These homologs bind APC2 in a manner that is similar to the cellular APC11, but lack Ub ligase activity due to specific alterations in the RING-H2 domain [112]. Therefore, rather than mediating ubiquitylation of substrates, these homologs are competitive inhibitors of APC/C function, and in agreement with that, their expression results in accumulation of APC/C substrates and cell cycle alterations [112,113]. Importantly, an engineered ORF virus lacking gene 014 (also known as poxvirus APC/cyclosome regulator or PACR) showed reduced viral replication, presumably because of the inability to exploit the cellular resources that accumulate in S-phase due to APC/C inhibition [112,114]. Supporting this idea, the poxvirus groups proficient for PACR are deficient for important viral enzymes contributing to the nucleotide pool such thymidine kinase and ribonucleotide reductase, which may explain why PACR homologs are not found in other poxvirus groups.

## 5. Poxvirus-Encoded E3 Ub Ligases and Ub Genes

Besides adaptors, E3 Ub ligases have also been identified in poxvirus genomes. This essentially involves two families of proteins: the poxvirus MARCH E3 Ub ligase and p28. The poxvirus MARCH (from membrane-associated RING-CH) consists of a RING-CH domain followed by two transmembrane domains, in an arrangement equivalent to that observed in the Kaposi’s sarcoma associated virus (KSHV) K3 and K5 Ub ligases [115]. It can be found in certain poxvirus species such as MYXV, swinepox virus, or lumpy skin disease virus [115]. The presence of transmembrane domains facilitates access to cellular membrane proteins, which can then be downregulated. Studies with the rabbit-specific MYXV have shown that its MARCH Ub ligase M153R contributes to virulence and is able to downregulate several plasma membrane proteins, including the major histocompatibility complex class I (MHC-I), the pro-apoptotic receptor Fas, and CD4 [116,117,118,119,120]. Downregulation of MHC-I by MYXV is reminiscent of that by KSHV MARCH proteins K3/K5 [115,121,122,123]. Mechanistically, M153R ubiquitylates a lysine residue in the cytosolic tail of MHC-I or CD4 and induces its internalisation and degradation through the lysosomal pathway [118]. This process results in enhanced susceptibility to natural killer (NK) cell lysis, a phenomenon that can be harnessed for MYXV immunotherapy [124,125].

The p28 Ub ligase is a viral protein containing N-terminal KilA-N domains followed by a C-terminal RING domain. It is present in multiple poxvirus genera, including VARV and several OPXV, although it is inactivated or absent in VACV strains widely used for experimentation, such as Western Reserve or Copenhagen [126,127]. In some cases, the gene appears as KilA-N domains without the C-terminal RING domain. Because of the similarities between the RING domain of p28 and a cellular protein termed Makorin, it was proposed that p28 generated as a fusion event following capture of *makorin* cDNA [128]. Early work with the rodent-specific ectromelia virus (ECTV) demonstrated the importance of p28 for viral replication in macrophages and its significant contribution to virulence [127,129]. Subsequently, p28 was shown to have Ub ligase activity, acting in concert with different E2 conjugating enzymes and targeting Ub to viral replication factories [130,131]. This localisation was dependent on the KilA-N domains, which were predicted and shown to bind DNA [131,132,133]. These observations strongly suggest that p28 ubiquitylates cellular (or viral) substrates. The identity of such substrates remains unknown. Several studies have linked p28 with inhibition of apoptosis, a response that is particularly strong in macrophages, where p28 is essential for viral replication [132,133,134]. This suggests that p28 counteracts a macrophage-specific cellular response that results in caspase-3 activation and apoptosis and virus attenuation in vivo. Identification of this response will yield important insights into host restriction of poxvirus replication.

Besides E3 Ub ligases, bioinformatics searches have also identified Ub-like genes in the genomes of certain avipoxvirus species, such as canarypox virus, penguinpox virus, or flamingopox virus, and several entomopoxviruses [135,136,137,138,139]. These viral Ub genes are >85% identical to human Ub, containing all the residues and features required for ubiquitylation, and are reminiscent of the Ub genes identified in baculoviruses, a family of dsDNA viruses that infect insects and share biological niche with entomopoxviruses. The function of this viral Ub genes remains to be investigated.

## 6. Ubiquitylation of Viral Proteins

Besides rewiring cellular E3 Ub ligases and targeting of cellular factors, viral proteins are also ubiquitylated during infection. As indicated above, poxvirus uncoating requires active UPS to mediate the breakdown of the internalised cores, which is consistent with the detection of K48-linked ubiquitylated cores [22]. Consistent with this idea, a global ubiquitylation analysis of CPXV virions has reported the predominant presence of K48-linked Ub chains in purified virions [140]. Intriguingly, this same study did not identify proteasomal degradation of core proteins, but of the uncoating factor D5 instead, which may indicate that D5 turnover is part of the uncoating process [140]. Besides D5, this global analysis identified >50 viral proteins that were degraded by the proteasome early in infection and >130 ubiquitylation sites matching to >50 different viral proteins, altogether impacting on a variety of processes including virus/host interactions, transcription, DNA replication, or morphogenesis [140]. In some cases, evidence of ubiquitylation and degradation of some of these proteins had already been reported by previous studies using VACV. For instance, protein F17 is a highly conserved structural protein and one of the most abundant components of the virion lateral bodies [141]. F17 is known to be degraded in a proteasome-dependent manner early in infection, and this process is thought to aid in the dismantling the lateral bodies and releasing enzymes contained therein [142]. One of these is the viral phosphatase VH1, known to mediate the dephosphorylation and inactivation of STAT1 [143], thereby showing that lateral bodies strategically package and deliver host modulators upon virion internalisation [144].

Some viral immunomodulators are also known to be directly ubiquitylated. Protein E3 is a dsRNA-binding protein known to prevent activation of protein kinase R and RNAse L and IFN-primed necroptosis [145,146,147,148], thus harbouring critical immune evasion functions. E3 was shown to be modified by SUMO and Ub, and these modifications affected the stability of the protein [149]. Similarly, protein N1 was shown to be ubiquitylated at multiple lysine sites [150]. N1 is a multifunctional Bcl-2-like protein that modulates cell death and inflammatory signalling [151,152,153,154]. Whilst ubiquitylation did not render the protein more susceptible for degradation [150], it is unclear whether it affected any of its multiple reported functions. Interestingly, other viral Bcl-2-like proteins, including C6, were shown not to be ubiquitylated but to indirectly associate with Ub [150], in line with the ability of C6 to induce HDAC proteasomal degradation (discussed below). An important question that remains to be addressed in all these cases is how these viral proteins are ubiquitylated and what Ub ligases are responsible for these events. The answers to these questions will not only expand our understanding of the interplay between poxviruses and the Ub system, but also provide new therapeutic targets for antiviral intervention.

## 7. Other Interactions with the Ub System

Recent proteomics work has shown that VACV infection of human fibroblasts results in the downregulation of 265 cellular proteins [155]. Out of these, ~70% were rescued by the proteasomal inhibitor MG-132, thereby demonstrating a direct involvement of the UPS. VACV degrades several groups of cellular ligands, such as the ephrin receptors and collagens, and ISGs, such as IFITs and tripartite motif containing proteins (TRIMs). How VACV achieves downregulation of all these cellular proteins is largely unknown. However, this has been discovered in some cases. Degradation of IFITs is mediated by the ANK/F-box protein C9 as indicated above [72]. Equally, the degradation of the histone deacetylase (HDAC) 4 and 5 is known to be mediated by the viral protein C6 [155,156], a multifunctional protein also known to target the IFN-responsive factor (IRF3) pathway to suppress the induction of IFN-I [157] as well as the Janus kinases–signal transducer and activator of transcription proteins (JAK–STAT) pathway to suppress the antiviral effects of IFN-I [158]. In agreement with these important functions, deletion of C6 attenuates infection and significantly improves the immunogenicity of VACV-derived vaccine vectors [101,102,157,159,160,161]. Both HDAC4 and HDAC5 are antiviral factors restricting VACV and herpes simplex virus type 1 infection, and this restriction is alleviated when C6 is expressed [155,156]. C6 has no discernible sequence indicating Ub ligase activity or association with the Ub system, so how C6 mediates proteasomal degradation of these HDACs remains to be elucidated.

## 8. Concluding Remarks

Over the last decades, Ub and ubiquitylation have emerged from being a cryptic little protein modification into a master regulator of cell biology. Our knowledge on how the Ub system interplays with poxviruses has also expanded accordingly. It is now clear that poxviruses encode many different types of molecules interacting with the Ub system. Many of these have been identified as adaptors of cellular E3 Ub ligases due to sequence similarity to their cellular counterparts. Whilst great progress has been made on identifying these adaptors and understanding how they operate, elucidating the substrates that are targeted by these virally assembled ligases remains an important task, as these targets are likely to be important restriction factors and antiviral molecules. Comparative virology and genetic approaches have proven useful, such as in the case of RIPK3 and, likewise, quantitative proteomics has allowed identification of the IFITs and HDAC4/5. Furthermore, complete proteome analyses have shown enormous potential in revealing important classes of cellular proteins downregulated (if eventually degraded) or modified during infection. These datasets uncover the vast reconfiguration that a cell encounters upon poxvirus infection, and they will shed light onto novel cellular hubs and functions in cell biology and immunity. Whilst ubiquitylation during viral infection is likely to be largely modulated by the many virally encoded Ub adaptors reported so far, it is important to consider the virally encoded E3 Ub ligases and the existence of yet unidentified non-canonical adaptors. Finally, it is now also clear that the UPS plays a major role in allowing uncoating, genome release, and allowing productive infection. Understanding how cellular proteasomes are recruited to the incoming viral cores and how they recognise core proteins are important questions because of the potential to yield new therapeutic approaches for antiviral intervention. Although poxviruses are the only DNA viruses to replicate exclusively in the cytosol, most RNA viruses uncoat and release their RNA genomes in the cytosol. Ubiquitylation is also involved in poxvirus DNA replication, and similar biological processes may be operating in the replication of other large DNA viruses such as African swine fever virus or the Herpesviridae family. Therefore, knowledge obtained using tractable poxvirus models has the potential to enlighten conserved mechanisms used by many viruses to establish infection. With the emergence of induced proximity drugs and related technologies, expanding and deepening our knowledge on virus interactions with the Ub system is a timely and promising avenue to contribute to the fight against transmissible and non-transmissible diseases that continue to be major causes of disability and death.

## Figures and Tables

**Table 1:** Conservation of ANK protein orthologues within OPXV.

Orthologue Group ^1^	CPXV (BR)	VACV (Cop)	VARV (B75)	ECTV (Mos)	MPXV (Z)	CMPV (CMS)
I	006/225	C19L	G1R	002	J1R	003L
II	008/223	C17L	D1L			004L
III	011			005		
IV	016			010		
V	017				D1L	
VI	019					
VII	025		D8L		D7L	
VIII	027	C9L			D9L	
IX	039	M1L	O1L	021	O1L	
X	041	K1L	C1L	022	C1L	
XI	198	B4R	B5R	154	B5R	177R
XII	200	B6R				
XIII	211	B18R	B16R	165	B17R	197R
XIV	213		B18R			
XV	220					

^1^ ANK orthologue groups were based on [37]. Complete genome accession numbers were as follows: CPXV Brighton-Red (BR), NC_003663; VACV Copenhagen (COP), M35027; VARV Bangladesh-1975 (B75), L22579; ECTV Moscow (Mos), NC_004105; MPXV Zaire (Z), NC_003310; Camelpox virus CMS, AY009089.

**Table 2:** Orthologues of BTB proteins within OPXV.

CPXV (GRI).	VACV (Cop)	ECTV (Mos)	MPXV (Z)	CMPV (CMS)
A57R	A55	150		172R
C18L	C2	018		24L
G3L	F3	027	C9L	38L
D11L				
B19R				
B9R		167 (C13R)		186R
	C5		D12L	21L

Complete genome accession numbers were as in Table 1. Complete genome accession numbers were as follows: CPXV GRI, X94355; VACV COP, M35027; ECTV Moscow (Mos), NC_004105; MPXV Zaire (Z), NC_003310; Camelpox virus CMS, AY009089.
